# Infant emergency department visits, readmission, and mortality by maternal anxiety disorder during pregnancy occurring with and without other mental health conditions: a retrospective cohort study

**DOI:** 10.1186/s12884-025-08603-y

**Published:** 2025-12-23

**Authors:** Rebecca J. Baer, Scott P. Oltman, Deborah Adeyemi, Ribka Amsalu, Kacie C. A. Blackman, Bridgette Blebu, Kimberly Coleman-Phox, Jennifer N. Felder, Dawn Gano, Audrey Lyndon, Safyer McKenzie-Sampson, Carolyn Ponting, Larry Rand, Elizabeth E. Rogers, Kelli K. Ryckman, Martina A. Steurer, Akila Subramaniam, Kelly D. Taylor, Karen M. Tabb, Laura Jelliffe-Pawlowski

**Affiliations:** 1 Department of Pediatrics, University of California San Diego, 9500 Gilman Drive MC0828, La Jolla, CA 92093 USA; 2 Department of Obstetrics, Gynecology and Reproductive Sciences, University of California, San Francisco, San Francisco, CA USA; 3 The California Preterm Birth Initiative, University of California San Francisco, San Francisco, CA USA; 4 The International Healthy Outcomes of Pregnancy for Everyone (HOPE) Research Consortium, University of California San Francisco, San Francisco, CA USA; 5 Department of Epidemiology and Biostatistics, University of California San Francisco, San Francisco, CA USA; 6 Institute of Global Health Sciences, University of California San Francisco, San Francisco, CA USA; 7 Department of Health Sciences, Health Equity Research and Education Center, California State University Northridge, Northridge, CA USA; 8 Department of Obstetrics and Gynecology, Lundquist Institute for Biomedical Innovation at Harbor-UCLA Medical Center, University of California, Los Angeles, Los Angeles, CA USA; 9 Osher Center for Integrative Health, University of California, San Francisco, San Francisco, CA USA; 10 Department of Psychiatry and Behavioral Sciences, University of California, San Francisco, San Francisco, CA USA; 11 Department of Neurology, University of California, San Francisco, San Francisco, CA USA; 12 Department of Pediatrics, University of California San Francisco, San Francisco, CA USA; 13 Rory Meyers College of Nursing, New York University, New York, NY USA; 14 Department of Pediatrics, Stanford University School of Medicine, Palo Alto, CA USA; 15 Department of Epidemiology and Biostatistics, Indiana University School of Public Health - Bloomington, Bloomington, IN USA; 16 Division of Maternal Fetal Medicine, Department of Obstetrics and Gynecology, University of Alabama at Birmingham, Birmingham, AL USA; 17 Division of Prevention Science, University of California San Francisco, San Francisco, CA USA; 18 School of Social Work, University of Illinois at Urbana-Champaign, Urbana, IL USA

**Keywords:** Anxiety, Depression, Mental health disorder, Adverse outcome, Infant, Emergency department, Readmission, Infant death

## Abstract

**Background:**

While a link between maternal anxiety diagnoses and adverse maternal and infant outcomes has been reported, there is a paucity of data regarding infant outcomes through the first year of life in those born to individuals with anxiety only and anxiety comorbid with other mental health conditions.

**Method:**

The sample included 5,836,541 singleton liveborn infants in California from 2007–2020. Anxiety with and without depression or a non-depression mental health condition during pregnancy were identified from ICD codes from hospital discharge records. Adverse infant outcomes evaluated included emergency department (ED) visit, readmission, or death in the first year of life. Log-linear regression was used to calculate the crude (cRR) and adjusted (aRR) relative risk and 95% confidence interval (CI) of each outcome by mental health condition grouping. To fully consider the potential influence of co-variants, we examined four different adjusted risk calculations. Patterns of comorbidity and infant outcomes were also examined within payer for birth (as an indicator of income) and racial/ethnic groupings.

**Results:**

Infants of birthing people diagnosed with anxiety alone or anxiety and depression had reduced ED visits (cRRs 0.93 and 0.85, respectively) and readmissions (cRRs 0.85 and 0.83) but an increased risk of death (cRRs 1.40 and 1.75) compared to those without any mental health condition. Infants of people with diagnoses of anxiety and other non-depression conditions during pregnancy faced higher risks across all metrics (ED visits and readmissions cRRs 1.14–1.20, death cRRs 2.07–3.81). Observed were often tempered in fully adjusted models.

**Conclusions:**

Maternal anxiety diagnosis during pregnancy was associated with an elevated risk of infant death and was exacerbated by the presence of another mental health condition. These relationships were not explained by the presence of other related risks. These findings underscore the need for interventions for birthing people with anxiety, and particularly for those with comorbid mental health conditions.

**Supplementary Information:**

The online version contains supplementary material available at 10.1186/s12884-025-08603-y.

## Background

Maternal anxiety is common, with approximately 15% of pregnant people receiving a diagnosis of anxiety during pregnancy, and its association with adverse birth outcomes is well-documented [1, 2]. Data have shown that pregnant people with anxiety during pregnancy are at increased risk for adverse birth outcomes like preterm birth (PTB, birth < 37 completed weeks of gestation), low birth weight (LBW, birthweight < 2500 g), small for gestational age (SGA, birthweight for sex and gestational age < 10th percentile), and infant mortality [3–10]. Maternal diagnosis of anxiety during pregnancy has also been associated with long-term neurodevelopmental delays, behavioral and emotional difficulties, impaired bonding, and an increased risk for illness and death in infants [8, 10–12]. The occurrence of other mental health conditions (e.g. depression, schizophrenia, bipolar disorder) during pregnancy are also related to an increased risk of adverse birth and infant outcomes [4, 8, 9, 13–20]. 

While a link between maternal anxiety diagnoses and maternal and infant outcomes during the first year of life has been reported [8, 10, 13, 14], there is a paucity of data regarding infant outcomes in those born to individuals with anxiety with or without comorbid mental health conditions. This is especially true with respect to diagnostic patterns among vulnerable populations, including racially/ethnically marginalized or low-income groups, who are known to be at increased risk for experiencing anxiety during pregnancy and for having adverse maternal and infant outcomes in the year after birth [2, 4, 8]. Further, understanding whether having a comorbid mental health condition is uniquely related to an increased risk in infants is critical for designing personalized care plans and for better understanding the causal underpinnings of anxiety and adverse infant outcome relationships. It is also critical to evaluate these relationships while considering other maternal and infant factors (e.g. PTB, SGA) given established associations with maternal anxiety and with infant outcomes [3–6, 8, 21, 22].

Here, we examine the risk of infant readmission, emergency room visits, and death in the first year of life among those born to pregnant individuals with an anxiety disorder alone, an anxiety disorder with depression, an anxiety disorder with a non-depression mental health condition, and among individuals with co-occurring anxiety disorder, depression, and another mental health condition during pregnancy. To fully consider the potential influence of co-variants, we examined sequentially adjusted risk calculations. Patterns of comorbidity and infant outcomes were also examined within payer for birth (as an indicator of income) and racial/ethnic groupings.

## Materials/subjects and methods

### Sample

**Fig. 1:**
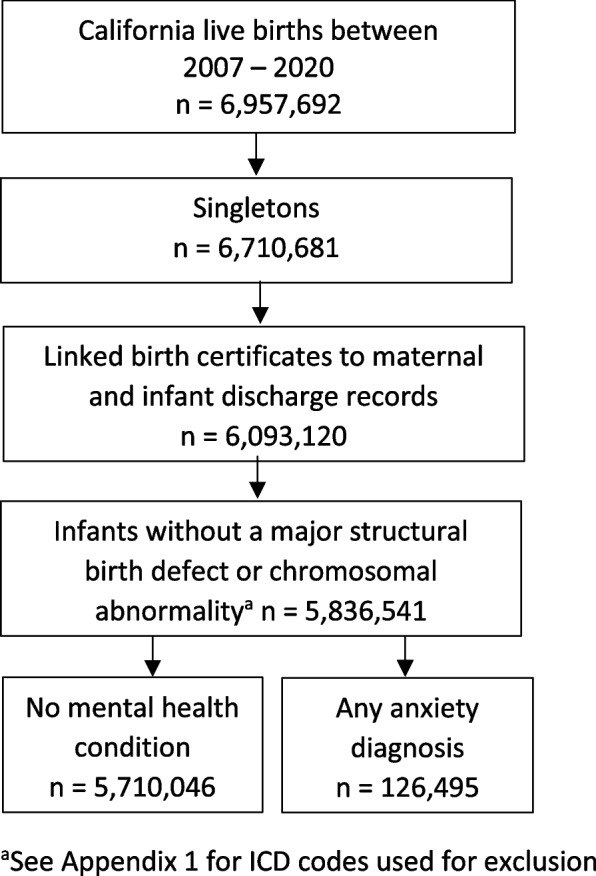
Sample selection

The sample was drawn from all California live-born infants between 2007 and 2020. Birth certificates, maintained by California Vital Statistics, were linked to hospital discharge, emergency department (ED), and ambulatory surgery records maintained by the California Department of Health Care Access and Information (HCAI) [23]. Hospital discharge, ED, and ambulatory surgery files provided diagnoses and procedure codes based on the International Classification of Diseases (ICD), as reported to HCAI by the healthcare facilities [24, 25]. The study sample was restricted to non-anomalous singletons with linked birth records for the birthing person and infant (Fig. 1).

#### Anxiety and other mental health conditions

Mental health diagnosis during pregnancy was identified by the presence of a diagnostic code in any HCAI record during pregnancy or for the birth admission. Mental health conditions were grouped as anxiety disorder alone, anxiety and depression, anxiety and other mental health diagnosis (e.g. schizophrenia, bipolar disorder, personality disorder), and anxiety, depression, and other mental health diagnosis (Appendix 1). The reference population was birthing people without any mental health diagnosis on a pregnancy or birth HCAI record. People who had a mental health diagnosis without a diagnosis of anxiety disorder were excluded from the study, for an analytical sample of 126,495 birthing people with an anxiety diagnosis and 5,710,046 birthing people with no mental health diagnosis (Fig. 1).

### Infant outcomes

Infant outcomes in the first year of life included an ED visit, hospital readmission, and/or death. ED visits and readmissions were captured from HCAI records. Death of a liveborn infant was obtained from vital statistics and/or HCAI records (discharge status = died). Final cause of death was reported by California Vital Statistics as a single ICD-10 code for all study years. Cause of death was grouped as perinatal complications, sudden unexpected infant death, non-accidental trauma, and other ICD-10 diagnoses (Appendix 1). Selection of these cause of death groups follows previous literature [8] and aimed to allow large enough cell sizes as well as clinically meaningful groups.

#### Infant and maternal covariates

Additional infant and maternal covariates were also considered. Gestational age and PTB (< 37 weeks gestation) were determined by the best obstetric estimate from birth certificate records. SGA was defined as a birthweight below the 10th percentile for gestational age and sex [26] using data from birth certificate records. Sociodemographic factors for birthing people included reported racial/ethnic group, education level, expected payer for birth, participation in the Special Supplemental Nutrition Program for Women, Infants, and Children (WIC) [27], and adequacy of prenatal care (calculated using gestational age at delivery, month of gestation at first prenatal care visit, and number of prenatal care visits) [28, 29] and were obtained from birth certificate records. Racial/ethnic group associations were calculated as being part of a group versus not to assess how patterns within a specific group compared to the rest of the population. Maternal health conditions and exposures included diabetes (preexisting or gestational), hypertension (preexisting or gestational), smoking during pregnancy (reported as number of cigarettes by trimester on birth certificate records), and drug or alcohol use during pregnancy. These variables were obtained from HCAI and birth certificate records (Appendix 1). Other maternal covariates included maternal age at the time of birth and parity, recorded in birth certificate records.

#### Statistical analysis

The risk of infant ED visit, readmission, or death was estimated by PTB, SGA, socio-demographic factors, age and parity, and maternal health conditions and exposures using Poisson log-linear regression with no adverse infant outcome as the reference population.

The risk of anxiety disorder overall and by subgroups of other mental health conditions was estimated by PTB, SGA, sociodemographic factors, maternal age, parity, health conditions, and exposures using Poisson log-linear regression with no adverse infant outcome as the reference population.

Estimates of the relative risk (RR) of adverse infant outcomes and 95% confidence interval (CI) were calculated sequentially using Poisson log-linear regression. For model 1, the unadjusted risk was calculated for each adverse infant outcome. For the adjusted models, the entrance of a potential covariate into any model required a significant (*p* < 0.05) association between anxiety disorder and the potential covariates. Model 2 included adjustment for PTB. Model 3 included adjustment for PTB, significant maternal sociodemographic factors, maternal age at delivery, and parity. Model 4 included all factors in the third model plus significant health conditions and exposures for the birthing person. The risk was also examined within payer (public or non-public) and racial/ethnicity grouping (Asian, Black, Hispanic, Other (includes American Indian/Alaska Native, Native Hawaiian/Pacific Islander, “Other race”, “multiracial” (two or more racial groups indicated), unknown/not stated), and by non-Hispanic White). Artificial intelligence (chatgpt.com) was used to create a figure displaying these relative risks.

Infant cause of death was examined across each maternal anxiety group and the risk of each cause of death was estimated in the same sequential fashion as the other adverse infant outcomes.

All analyses were performed using Statistical Analysis Software version 9.4 (SAS, Cary, NC). Methods and protocols for the study were approved by the Committee for the Protection of Human Subjects within the Health and Human Services Agency of the State of California.

## Results

The sample included 5,836,541 births, of whom 126,495 (2.2%) had an anxiety disorder diagnosis during pregnancy or at birth as recorded in an HCAI record. Of the 126,495 birthing people with a diagnosis of an anxiety disorder, 89,297 (70.6%) had a documented anxiety disorder alone, 29,840 (23.6%) had a diagnosis of anxiety and depression (but no other mental health diagnosis), 5,357 (4.2%) had anxiety and a non-depression mental health diagnosis, and 2,001 (1.6%) had anxiety with depression and another mental health diagnosis. The sample was 50.2% Hispanic, 14.0% Asian, 4.9% Black, and 26.0% white, non-Hispanic. Over 50% (52.0%) had more than 12 years of education and 46.2% had public health insurance.

Risk factors for adverse infant outcomes included PTB, education < 12 years, public insurance, WIC participation, inadequate prenatal care, < 18 years of age at delivery, infection, smoking during pregnancy, and drug/alcohol use during pregnancy (Table 1). When compared to all other racial/ethnicity groups, Hispanic, Black, American Indian/Alaska Native, and Native Hawaiian/Pacific Islander groups were found to be more likely to have an infant with an ED visit, readmission, or death in the first year while Asian and White groups were less likely (Table 1).

**Table 1:** Association of infant and birthing person factors with adverse infant outcome

	**No ED Visit, readmission or death**	**ED visit**	**Readmission**	**Death**
	n (%)	n (%)	n (%)	n (%)
		cRR (95% CI)	cRR (95% CI)	cRR (95% CI)
Sample	3,815,284	1,722,714	548,636	17,076
Infant factors				
Preterm birth				
< 37 weeks	219,116 (5.7)	123,083 (7.1)	58,058 (10.8)	8,756 (51.3)
		1.2 (1.2, 1.2)	1.8 (1.8, 1.8)	18.2 (17.7, 18.8)
≥ 37 weeks	3,591,328 (94.1)	1,597,305 (92.7)	479,869 (89.1)	7,582 (44.4)
	Reference			
Small for gestational age^c^	318,448 (8.4)	153,702 (8.9)	49,779 (9.2)	2,039 (11.9)
		1.1 (1.0, 1.1)^a^	1.1 (1.1, 1.1)	1.5 (1.4, 1.6)
Birthing person socio-demographic factors				
Racial/ethnic group^c^				
Hispanic	1,741,941 (45.7)	1,041,710 (60.5)	296,945 (55.1)	8,591 (50.3)
		1.5 (1.5, 1.5)	1.4 (1.4, 1.4)	1.2 (1.2, 1.2)
Non-Hispanic				
Asian	632,715 (16.6)	136,795 (7.9)	63,090 (11.7)	1,750 (10.3)
		0.5 (0.5, 0.5)	0.7 (0.7, 0.7)	0.6 (0.5, 0.6)
Black	156,081 (4.1)	116,104 (6.7)	29,849 (5.5)	1,966 (11.5)
		1.4 (1.4, 1.4)	1.3 (1.3, 1.3)	3.0 (2.9, 3.2)
Other	197,813 (5.2)	74,443 (4.3)	25,488 (4.7)	1,268 (7.4)
		1.0 (1.0, 1.0)^a^	1.0 (1.0, 1.0)^a^	1.3 (1.2, 1.4)
American Indian/Alaska Native	11,902 (0.3)	6,059 (0.4)	1,870 (0.4)	83 (0.5)
		1.1 (1.1, 1.1)	1.1 (1.0, 1.1)^a^	1.6 (1.3, 1.9)
Native Hawaiian/Pacific Islander	13,950 (0.4)	7,612 (0.4)	2,583 (0.4)	110 (0.6)
		1.1 (1.1, 1.2)	1.3 (1.2, 1.3)	1.8 (1.5, 2.1)
“Other”	2,121 (0.1)	954 (0.1)	289 (0.1)	^d^
		1.0 (0.9, 1.1)	1.0 (0.9, 1.1)	1.1 (0.6, 2.0)
Multiracial	80,904 (2.1)	32,423 (1.9)	10,221 (1.9)	403 (2.4)
		0.9 (0.9, 0.9)	0.9 (0.9, 0.9)	1.1 (1.0, 1.2)^a^
White	1,086,734 (28.5)	353,661 (20.5)	123,264 (22.9)	3,501 (20.5)
		0.7 (0.7, 0.7)	0.8 (0.8, 0.8)	0.6 (0.6, 0.7)
Education (years)				
< 12	619,083 (16.2)	434,170 (25.2)	123,036 (22.8)	3,734 (21.9)
		1.1 (1.1, 1.1)	1.2 (1.1, 1.2)	1.1 (1.0, 1.1)^a^
12	861,472 (22.6)	515,706 (29.9)	144,395 (26.8)	4,867 (28.5)
	Reference			
> 12	2,167,378 (56.8)	707,381 (41.1)	248,643 (46.2)	7,013 (41.1)
		0.7 (0.7, 0.7)	0.7 (0.7, 0.7)	0.6 (0.6, 0.6)
Payer for birth				
Private	2,044,189 (53.6)	589,739 (34.2)	216,546 (40.2)	6,395 (37.5)
	Reference			
Public	1,507,414 (39.5)	1,053,869 (61.2)	294,394 (54.7)	9,497 (55.6)
		1.8 (1.8, 1.8)	1.7 (1.7, 1.7)	2.0 (1.9, 2.1)
TRICARE (Active Duty Military)	18,431 (0.5)	5,714 (0.3)	1,991 (0.4)	64 (0.4)
		1.1 (1.0, 1.1)^a^	1.0 (1.0, 1.1)	1.1 (0.9, 1.4)
Other	245,250 (6.4)	73,392 (4.3)	25,705 (4.8)	1,120 (6.6)
		1.03 (1.0, 1.0)^a^	1.0 (1.0, 1.0)	1.5 (1.4, 1.6)
WIC participation				
No	2,127,705 (55.8)	596,300 (34.6)	225,192 (41.8)	7,852 (46.0)
	Reference			
Yes	1,649,863 (43.2)	1,111,038 (64.5)	308,088 (57.2)	8,683 (50.9)
		1.8 (1.8, 1.8)	1.6 (1.6, 1.7)	1.4 (1.4, 1.5)
Adequacy of prenatal care^b^				
Adequate plus/adequate	2,761,556 (72.4)	1,218,460 (70.7)	389,150 (72.3)	9,745 (57.1)
	Reference			
Intermediate	534,618 (14.0)	237,599 (13.8)	70,504 (13.1)	1,688 (9.9)
		1.01 (1.0, 1.0)^a^	0.9 (0.9, 1.0)^a^	0.9 (0.9, 0.9)
Inadequate	374,881 (9.8)	199,258 (11.6)	57,399 (10.7)	2,423 (14.2)
		1.1 (1.1, 1.1)	1.1 (1.1, 1.1)	1.8 (1.7, 1.9)
Other birthing person factors				
Age at delivery (years)				
< 18	60,952 (1.6)	50,771 (3.0)	13,189 (2.5)	517 (3.0)
		1.4 (1.4, 1.4)	1.4 (1.4, 1.4)	1.8 (1.7, 2.0)
18–34	2,916,656 (76.5)	1,408,955 (81.8)	425,822 (79.1)	13,454 (78.8)
	Reference			
> 34	837,556 (22.0)	262,945 (15.3)	99,609 (18.5)	3,096 (18.1)
		0.7 (0.7, 0.7)	0.8 (0.8, 0.8)	0.8 (0.8, 0.8)
Parity				
Nulliparous	1,487,757 (39.0)	668,191 (38.8)	201,613 (37.4)	6,975 (40.9)
		0.99 (1.0, 1.0)^a^	0.9 (0.9, 0.9)	1.1 (1.0, 1.1)^a^
Multiparous	2,324,378 (60.9)	1,053,250 (61.1)	336,588 (62.5)	9,988 (58.5)
	Reference			
Health conditions/exposures				
Diabetes				
None	3,407,644 (89.3)	1,561,940 (90.7)	483,160 (89.7)	15,363 (90.0)
	Reference			
Gestational diabetes	367,170 (9.6)	144,966 (8.4)	48,723 (9.1)	1,341 (7.9)
		0.9 (0.9, 0.9)	0.9 (0.9, 1.0)^a^	0.8 (0.8, 0.9)
Pre-existing diabetes	40,470 (1.1)	15,808 (0.9)	6,753 (1.3)	372 (2.2)
		0.9 (0.9, 0.9)	1.2 (1.1, 1.2)	2.5 (2.3, 2.7)
Hypertension				
None	3,479,416 (91.2)	1,584,916 (92.0)	493,274 (91.6)	14,898 (87.3)
	Reference			
Gestational hypertension	117,996 (3.1)	45,433 (2.6)	13,989 (2.6)	407 (2.4)
		0.9 (0.9, 0.9)	0.9 (0.8, 0.9)	0.8 (0.7, 0.9)
Pre-existing hypertension	50,355 (1.3)	20,674 (1.2)	6,967 (1.3)	464 (2.7)
		0.9 (0.9, 0.9)	1.0 (1.0, 1.0)	2.1 (2.0, 2.3)
Preeclampsia	147,110 (3.9)	62,384 (3.6)	21,545 (4.0)	1,157 (6.8)
		0.96 (1.0, 1.0)^a^	1.0 (1.0, 1.1)^a^	1.9 (1.8, 2.0)
Infection^c^	309,394 (8.1)	188,580 (11.0)	55,501 (10.3)	2,771 (16.2)
		1.2 (1.2, 1.3)	1.3 (1.2, 1.3)	2.2 (2.1, 2.3)
Smoked during pregnancy^c^	72,455 (1.9)	52,686 (3.1)	14,252 (2.7)	1,004 (5.9)
		1.4 (1.4, 1.4)	1.3 (1.3, 1.4)	3.2 (3.0, 3.4)
Drug/alcohol use during pregnancy^c^	60,351 (1.6)	34,207 (2.0)	11,392 (2.1)	939 (5.5)
		1.2 (1.2, 1.2)	1.3 (1.3, 1.3)	3.6 (3.4, 3.8)

*WIC* Special Supplemental Nutrition Program for Women, Infants, and Children, *cRR* unadjusted relative risk, *CI* confidence interval

^a^*p* < 0.05

^b^Based on Kotelcheck and colleagues [29]

^c^versus no

^d^n < 11

A higher proportion of people with a diagnosis of anxiety during pregnancy were self-reported Non-Hispanic Black, American Indian/Alaska Native, multi-racial, or White compared to birthing people without a mental health diagnosis. The majority of people with or without an anxiety disorder had an education > 12 years, have private insurance, and had adequate prenatal care, although these rates were higher among people with a diagnosis of anxiety. People with an anxiety disorder during pregnancy were more likely to have a PTB, have gestational diabetes, preexisting diabetes, gestational hypertension, preexisting hypertension, preeclampsia, infection, smoked during pregnancy, and used drugs/alcohol during pregnancy compared to people without a mental health diagnosis. These patterns were generally consistent across subgroups with anxiety (anxiety alone, anxiety and depression, anxiety and non-depression mental health diagnosis, or anxiety, depression, and other mental health diagnosis) (Supplemental Table 1).

In unadjusted models, birthing people with an anxiety disorder alone, or anxiety and depression only were less likely to have an infant with an ED visit (crude relative risks (cRRs) 0.85–0.93) or readmission in the first year of life (cRR 0.83–0.85) but were more likely to experience an infant death (cRR 1.50–1.75) compared to birthing people without a mental health diagnosis. In the fully adjusted model, birthing people with anxiety alone or anxiety and depression were less likely to have an infant with a readmission (adjusted risk ratios (aRRs) 0.87 and 0.81, respectively), and those with anxiety and depression were less likely to have an infant with an ED visit (aRR 0.92).

**Fig. 2:**
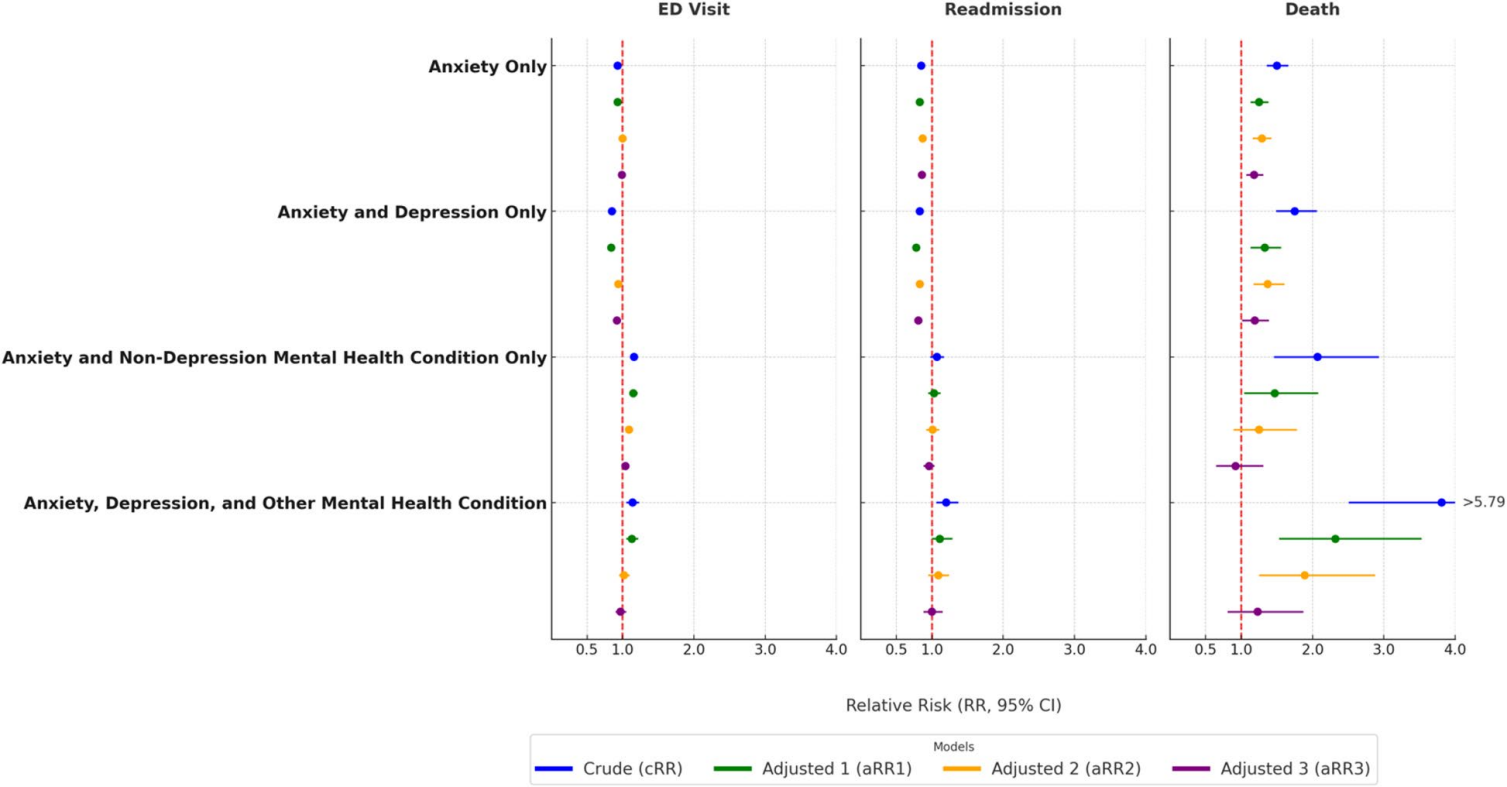
Relative Risk of adverse infant outcome by maternal anxiety diagnosis during pregnancy with or without comorbid mental health diagnosis, cRR: unadjusted relative risk. aRR1: adjusted for preterm birth. aRR2: adjusted for preterm birth, racial/ethnicity group, education, payer (when applicable), WIC participation, adequacy of prenatal care, birthing person age, and parity. aRR3: adjusted for preterm birth, racial/ethnicity group, education, payer (when applicable), WIC participation, adequacy of prenatal care, birthing person age, parity, gestational diabetes, gestational diabetes, preexisting HTN, gestational HTN, preeclampsia, infection, smoking, drug/alcohol use diagnosis

The risk of infant death was 1.18 to 1.19 in the final adjusted model for people with anxiety alone or anxiety and depression (Fig. 2, supplemental Table 2). In unadjusted models, birthing people with anxiety disorder and a non-depression mental health diagnosis were at increased risk of an infant with an ED visit (cRR 1.14 to 1.16) and infant death (cRR 2.07 to 3.81). The final adjusted model attenuated these risks (Fig. 2, supplemental Table 2).

Unadjusted risk of infant death due to perinatal complications and sudden unexpected infant death were elevated among people with an anxiety disorder during pregnancy (perinatal complications cRR 1.30 to 5.25; sudden unexpected infant death cRR 1.52 to 3.70) compared to people without a mental health diagnosis. The risk of sudden unexpected infant death remained elevated among people with an anxiety disorder during pregnancy for the first two adjusted models, but were no longer significant in the final model. The sample size was too small to examine cause of death across subgroups of anxiety diagnoses separately, but together these infants were at an elevated risk of death due to perinatal complications in all models (cRR 5.25, final model aRR 1.77) (Table 2).

**Table 2:** Cause of infant death by anxiety disorder

	**Without mental health diagnosis**	**Anxiety only**	**Anxiety and depression only**	**Anxiety and non-depression mental health disorder only**	**Anxiety, depression, and other diagnosis**
	n (%)	n (%)	n (%)	n (%)	n (%)
		cRR (95% CI)	cRR (95% CI)	cRR (95% CI)	cRR (95% CI)
		aRR1 (95% CI)	aRR1 (95% CI)	aRR1 (95% CI)	aRR1 (95% CI)
		aRR2 (95% CI)	aRR2 (95% CI)	aRR2 (95% CI)	aRR2 (95% CI)
		aRR3 (95% CI)	aRR3 (95% CI)	aRR3 (95% CI)	aRR3 (95% CI)
Survived to 1 year	5,710,046	89,297	29,840	5,357	2,001
	Reference				
Perinatal complications	8,168 (0.1)	166 (0.2)	64 (0.2)	15 (0.3)	15 (0.8)
		1.30 (1.12, 1.52)	1.50 (1.17, 1.92)	1.96 (1.18, 3.25)	5.25 (3.16, 8.71)
		0.97 (0.84, 1.14)	0.98 (0.77, 1.25)	1.17 (0.70, 1.93)	2.53 (1.52, 4.20)
		1.00 (0.85, 1.16)	1.02 (0.80, 1.30)	1.07 (0.65, 1.78)	2.39 (1.44, 3.97)
		0.93 (0.80, 1.09)	0.92 (0.72, 1.18)	0.87 (0.52, 1.45)	1.77 (1.06, 2.96)
Sudden unexpected infant death	2,312 (0.0)	55 (0.1)	24 (0.1)	^a^	^a^
		1.52 (1.17, 1.99)	1.99 (1.33, 2.97)	3.70 (1.85, 7.40)	^b^
		1.47 (1.13, 1.92)	1.88 (1.26, 2.81)	3.44 (1.72, 6.89)	^b^
		1.73 (1.32, 2.26)	2.24 (1.50, 3.36)	2.95 (1.47, 5.91)	^b^
		1.21 (0.92, 1.59)	1.25 (0.83, 1.87)	1.01 (0.50, 2.04)	^b^
Non-accidental trauma	238 (0.0)	^a^	^a^	^a^	^a^
		1.35 (0.55, 3.26)	^b^	^b^	^b^
		1.29 (0.53, 3.14)	^b^	^b^	^b^
		1.53 (0.63, 3.72)	^b^	^b^	^b^
		1.22 (0.50, 2.99)	^b^	^b^	^b^
Other	2,020 (0.0)	38 (0.0)	16 (0.1)	^a^	^a^
		1.20 (0.87, 1.66)	1.52 (0.93, 2.48)	2.65 (1.10, 6.36)	^b^
		1.07 (0.78, 1.48)	1.27 (1.78, 2.08)	2.11 (0.88, 5.06)	^b^
		1.17 (0.85, 1.62)	1.41 (0.86, 2.31)	1.94 (0.81, 4.67)	^b^
		0.99 (0.72, 1.37)	1.08 (0.66, 1.77)	1.09 (0.45, 2.64)	^b^

*cRR* unadjusted relative risk, *aRR1*: adjusted for preterm birth, *aRR2* adjusted for preterm birth, education, payer, WIC participation, adequacy of prenatal care, birthing person age, and parity, *aRR3* adjusted for preterm birth, education, payer, WIC participation, adequacy of prenatal care, birthing person age, parity, gestational diabetes, gestational diabetes, preexisting hypertension, gestational hypertension, preeclampsia, infection, smoking, drug/alcohol use diagnosis

^a^not displayed when n < 11

^b^not calculated when n < 5

Patterns of infant outcome risk stratified by anxiety group, payer, and racial/ethnic group were similar in magnitude, although they were often not found to be significant at *p* < 0.05 due, in part, to sample size. A notable difference was that infants born to those with anxiety alone and public health insurance were at slightly increased risk of an ED visit in all models. Unadjusted risk of infant death was highest among people with anxiety, depression, and another mental health diagnosis during pregnancy. Generally, people with anxiety alone or anxiety and depression were less likely to have an infant readmitted to the hospital during the first year of life (Fig. 2, supplemental Table 2, supplemental Table 3).

## Discussion

Using a large population-based California sample, we found that birthing people with an anxiety disorder diagnosis alone were less likely to have an infant with an ED visit or readmission during the first year of life, but were at higher risk of experiencing an infant death compared to people without a documented mental health diagnosis during pregnancy. In contrast, those with anxiety and a non-depression mental health diagnosis were more likely to have an infant with an ED visit or readmission during the first year of life, and were at an unadjusted 2.1 to 3.8-fold higher risk of experiencing an infant death. These associations held after adjusting for infant and maternal factors.

For all groups with an anxiety disorder, patterns by payer for delivery and racial/ethnic group varied, but general findings matched the total sample results, with the exception of people with anxiety alone and public health insurance being at slightly increased risk of an infant with an ED visit. People with an anxiety disorder were more likely to experience an infant death due to perinatal complications and sudden unexpected infant death. These risks, however, were no longer significant in the fully adjusted model, indicating that the association was confounded by factors not related to mental health.

### Results in context of what is known

Maternal anxiety during pregnancy has been associated with PTB, LBW, and SGA at birth [3–7], and for adverse childhood outcomes like neurodevelopmental, behavioral, and emotional challenges [11, 12]. Other mental health conditions, such as depression, have also been shown to be associated with adverse infant outcomes (e.g. preterm birth, low birthweight). Notably, we previously demonstrated that people with a mental health disorder during pregnancy (including anxiety disorders) were at increased risk of having an infant with a visit to the ED, readmission, or death in the first year of life in a similar population-based sample but we did not examine outcomes by patterns of co-occurring mental health conditions [8].

The findings in this paper further evaluate infant health indicators among people with anxiety disorders during pregnancy. We found that infants born to people with a diagnosed anxiety disorder alone or anxiety with depression were less likely to have an ED visit or readmission but more likely to experience an infant death. These findings align with others and may highlight opportunities for interventions that improve access to resources and timely care among newborn parents with mental health conditions [30, 31].

Importantly, we made adjustments in a progressive fashion that demonstrated that infant PTB, in particular, affected the observed association between anxiety grouping and infant outcomes. This observed relationship was further supported by the findings related to the cause of infant death. Infants born to people with a diagnosis of anxiety were more likely to have a final cause of death of perinatal complications or sudden unexpected infant death, which have both been shown to be more common in infants with preterm birth [32–35]. In the final adjusted model, the risk of sudden unexpected infant death was no longer significant. Although these associations are important, the unadjusted risk of adverse outcomes is also relevant in a clinical setting. Notably, these findings align with those of previous investigators [8–10, 36].

### Clinical implications

This study highlights the vulnerability of infants born to people with anxiety disorders during pregnancy. Detection and treatment of mental health conditions during pregnancy and, importantly, support for parents within the year after birth may help mitigate adverse impacts [37]. Mental health conditions are underdiagnosed and undertreated during pregnancy [38–40]. Data indicate that racially/ethnically marginalized groups and people with low income are especially less likely to receive a diagnosis or treatment [41–43]. There are promising and efficacious treatments for anxiety during pregnancy, and more studies are needed examining how to implement these into clinical and community practices in general and equitably [38, 44–48].

### Research implications

This study points to the importance of further investigation into potentially causal mechanisms for observed patterns particularly as they relate to the increased risk observed for infant death when anxiety occurs in isolation compared to when it co-occurs with depression and/or with other mental health conditions. While it is known that perinatal stress, immune, endocrine, microbiome, and placental-related factor patterns differ in people with mental health conditions and their infants and that such patterning is associated with adverse birth and infant outcomes [21, 49–59], it is not known whether observed patterns differ by the co-occurrence of conditions. Better understanding of these patterns could be key to unlocking novel interventions and for identifying pregnant individuals and infants at increased risk.

### Strengths and limitations

This study is unique because it uses a large population-based sample to evaluate the risk of key health indicators during the first year of life for an infant. The dataset used has information regarding important comorbidities and infant factors that affected observed relationships. The sample is socially diverse and we are able to provide information about patterning by payer and by racial/ethnic group, although sample sizes became small for infant death. This study, however, is not without limitations. Most notably, we identified anxiety disorders from hospital discharge records and our ascertainment was approximately 2% of the population, which is lower than expected in the population. Anxiety disorder and other mental health conditions often go undiagnosed [38]. particularly in racial/ethnic minoritized, low-income, and non-English speaking individuals [60]. Additionally, clinical diagnoses of anxiety during pregnancy (the only indication available in this dataset) is often much lower than self-reported anxiety symptoms during pregnancy – particularly in minoritized racial/ethnic groups [2]. As such, it is important to recognize that this study represents an undercount of the burden of mental health conditions in general and especially in vulnerable subgroups. While we do not believe this underdiagnosis negates the study findings (as the inclusion of undiagnosed individuals in the unaffected comparison group would have pulled the RRs and their 95% CIs to the null), this likely underdiagnosis suggests that results regarding rates of disorders within groups should not be relied upon for public health planning and points to the importance of follow-up studies in clinical settings with universal screening.

Also of note is that using an administrative database limits the information we have about the diagnosis, such as length of time with anxiety, treatment, and whether the anxiety continued into the postpartum period. Measurement and consideration of these factors will also be key as this work progresses. We also lack information about health care utilization outside the ED or hospital setting. Consideration of this information will be important in future work.

## Conclusion

Anxiety diagnosis during pregnancy was found to elevate infant risk of death and was exacerbated by the presence of another mental health condition. Infants born to people with anxiety, depression, and other mental health conditions faced triple the risk of death in year one compared to those without anxiety or another mental health condition. Risk of adverse infant outcome was tempered in fully adjusted models suggesting that a multitude of factors influence observed patterns. Study insights underscore the need for stronger support for pregnant and postpartum parents with anxiety and more supportive interventions for their infants and families – overall and particularly, when the person is faced with comorbid mental health conditions.

## Supplementary Information

Supplementary Material 1.

Supplementary Material 2.

Supplementary Material 3.

Supplementary Material 4.

## Data Availability

The data that support the findings of this study are available from the California Department of Health Care Access and Information (https://hcai.ca.gov/data-and-reports/request-data/) and California Vital Statistics (https://www.cdph.ca.gov/Programs/CHSI/Pages/Data-Applications.aspx), but restrictions apply to the availability of these data, which were used under California Committee for the Protection of Human Subjects IRB for the current study, and so are not publicly available.

## Abbreviations

CI: 95% Confidence interval
cRR: Crude relative risk
ED: Emergency department
HCAI: California department of health care access and information
ICD: International classification of diseases
LBW: Low birth weight
PTB: Preterm birth
SAS: Statistical analysis software
SGA: Small for gestational age
WIC: Special supplemental nutrition program for women, infants, and children
