# Supplementary material for: Infant emergency department visits, readmission, and mortality by maternal anxiety disorder during pregnancy occurring with and without other mental health conditions: a retrospective cohort study
Source: BMC Pregnancy Childbirth. 2025 Dec 23;26:83. doi: 10.1186/s12884-025-08603-y (PMC12837128; doi:10.1186/s12884-025-08603-y)
Supplement: Supplementary file 1 — Supplementary Material 1. [file 12884_2025_8603_MOESM1_ESM.docx]

Appendix 1. International Classification of Diseases codes and indication of birth certificate ascertainment for sample variables

|  | ICD-9 | ICD-10 | Birth certificate |
| --- | --- | --- | --- |
| Sample exclusion variables |  |  |  |
| Infant major structural birth defect | 740.0, 740.1, 740.2, 741.0, 741.9, 742.0, 742.1, 742.2, 742.3, 743.0, 743.1, 743.3, 743.4, 745.0, 745.1, 745.2, 745.3, 745.6, 746.0, 746.1, 746.2, 746.3, 746.7, 747.1, 747.4, 748.0, 748.4, 748.5, 749, 750.3, 750.5, 751.1, 751.2, 751.3, 751.6, 752.6, 752.7, 753.0, 753.2, 753.5, 753.6, 755.2, 755.3, 756.0, 756.4, 756.5, 756.6, 756.7 | Q00, Q01, Q02, Q03, Q04.0, Q04.1, Q04.2, Q04.3, Q05, Q9, Q11, Q12.0, Q13, Q20.0, Q20.2, Q20.3, Q20.4, Q20.5, Q20.8, Q21.2, Q21.3, Q22.0, Q22.1, Q22.2, Q22.3, Q22.4, Q22.5, Q23.4, Q25.1, Q25.3, Q26, Q30.0, Q33, Q39.0, Q39.1, Q39.2, Q39.3, Q39.4, Q35, Q36, Q37, Q40.0, Q41, Q42, Q43.1, Q44, Q54, Q55, Q56, Q60, Q62, Q64.1, Q64.3, Q71, Q72, Q75, Q78, Q79.0, Q79.1, Q79.2, Q79.3, Q79.4, Q79.5 |  |
| Infant chromosomal abnormality | 758 | Q9 |  |
| Maternal variables |  |  |  |
| Anxiety | 300 | F4 |  |
| Depression | 296.2, 296.3, 311 | F32.0, F32.1, F32.2, F32.3, F32.8, F32.9, F33.0, F33.1, F33.2, F33.3, F33.4, F33.8, F33.9 |  |
| Other mental health diagnosis | 295, 296.0, 296.1, 296.4, 296.5, 296.6, 296.7, 296.8, 296.9, 297, 298, 299, 301, 302 | F2, F30, F31, F34, F38, F39, F4, F5, F6 |  |
| Gestational diabetes | 648.8, infant 775.0 | O24.4, infant P70.0 | X |
| Preexisting diabetes | 249, 250, 648.0, infant P70.1 | E10, E11, E12, E13, E14,O24.0, O24.1, O24.2, O24.3 | X |
| Gestational hypertension | 642.3, Infant 760.0 | O13, infant P00.0 | X |
| Preexisting hypertension | 642.0, 642.1, 642.2 | O10 | X |
| Smoking | 305.1, 649.0 | F17, O99.33 | X |
| Drug/alcohol use | 303, 304, 305.0, 305.2, 305.3, 305.4, 305.5, 305.6, 305.7, 305.8, 305.9, 648.3 | F10, F11, F12, F13, F14, F15, F16, F18, F19, O99.32 |  |
| Final causes of infant death^a^ |  |  |  |
| Perinatal cause |  | P |  |
| Sudden unexpected infant death |  | R95, R99, W75 |  |
| Non-accidental trauma |  | T74, T76, X9, Y0 |  |
| Birth defect/chromosomal abnormality |  | Q |  |

^a^cause of death only reported as ICD-10
