# Supplementary material for: Infant emergency department visits, readmission, and mortality by maternal anxiety disorder during pregnancy occurring with and without other mental health conditions: a retrospective cohort study
Source: BMC Pregnancy Childbirth. 2025 Dec 23;26:83. doi: 10.1186/s12884-025-08603-y (PMC12837128; doi:10.1186/s12884-025-08603-y)
Supplement: Supplementary file 2 — Supplementary Material 2. [file 12884_2025_8603_MOESM2_ESM.docx]

Supplemental Table 1. Association of infant and birthing person factors with mental health disorder diagnosis on hospital discharge record during pregnancy or at birth

|  | **Without mental health diagnosis** | **Any anxiety diagnosis** | **Anxiety only** | **Anxiety and depression only** | **Anxiety and non-depression mental health disorder only** | **Anxiety, depression, and other diagnosis** |
| --- | --- | --- | --- | --- | --- | --- |
|  | n (%) | n (%) | n (%) | n (%) | n (%) | n (%) |
|  | cRR | cRR | cRR | cRR | cRR | cRR |
| **Sample** | 5,710,046 | 126,495 | 89,297 | 29,840 | 5,357 | 2,001 |
| **Infant factors** |  |  |  |  |  |  |
| **Preterm birth** |  |  |  |  |  |  |
| Any <37 weeks | 368,438 (6.5) | 12,323 (9.7) | 8,178 (9.20 | 3,202 (10.7) | 641 (12.0) | 302 (15.1) |
|  |  | 1.5 (1.5, 1.6) | 1.5 (1.4, 1.5) | 1.7 (1.7, 1.8) | 2.0 (1.8, 2.1) | 2.6 (2.3, 2.9) |
| ≥37 weeks | 5,333,614 (93.4) | 114,026 (90.1) | 81,019 (90.7) | 26,612 (89.2) | 4,704 (87.8) | 1,691 (84.5) |
|  | Reference |  |  |  |  |  |
| **Small for gestational age^e^** | 488,369 (8.6) | 11,056 (8.7) | 7,622 (8.5) | 2,546 (8.5) | 616 (11.5) | 272 (13.6) |
|  |  | 1.02 (1.0, 1.0)^a^ | 1.0 (1.0, 1.0) | 1.0 (1.0, 1.0) | 1.4 (1.3, 1.5) | 1.7 (1.5, 1.9) |
| **Birthing person sociodemographic factors** |  |  |  |  |  |  |
| **Racial/ethnic group**^e^ |  |  |  |  |  |  |
| Hispanic | 2,883,225 (50.5) | 45,874 (36.3) | 33,596 (37.6) | 10,109 (33.9) | 1,542 (28.8) | 627 (31.3) |
|  |  | 0.6 (0.6, 0.6) | 0.6 (0.6, 0.6) | 0.5 (0.5, 0.5) | 0.4 (0.4, 0.4) | 0.4 (0.4, 0.5) |
| Non-Hispanic |  |  |  |  |  |  |
| Asian | 808,729 (14.2) | 6,682 (5.3) | 5,146 (5.8) | 1,367 (4.6) | 120 (2.2) | 49 (2.5) |
|  |  | 0.3 (0.3, 0.4) | 0.4 (0.4, 0.4) | 0.3 (0.3, 0.3) | 0.1 (0.1, 0.2) | 0.2 (0.1, 0.2) |
| Black | 279,149 (4.9) | 6,958 (5.5) | 4,625 (5.2) | 1,612 (5.4) | 477 (8.9) | 244 (12.2) |
|  |  | 1.1 (1.1, 1.2) | 1.1 (1.0, 1.1)^a^ | 1.1 (1.1, 1.2) | 1.9 (1.7, 2.1) | 2.7 (2.4, 3.1) |
| Other | 277,794 (4.9) | 10,337 (8.2) | 6,798 (7.6) | 2,701 (9.1) | 604 (11.3) | 234 (11.7) |
|  |  | 1.7 (1.6, 1.7) | 1.5 (1.5, 1.6) | 1.9 (1.8, 2.0) | 3.0 (2.7, 3.3) | 2.7 (2.3, 3.2) |
| American Indian/Alaska Native | 18,315 (0.3) | 669 (0.5) | 418 (0.5) | 176 (0.6) | 52 (1.0) | 23 (1.2) |
|  |  | 1.6 (1.5, 1.8) | 1.5 (1.3, 1.6) | 1.8 (1.6, 2.1) | 3.0 (2.3, 4.0) | 3.6 (2.4, 5.4) |
| Native Hawaiian/Pacific Islander | 22,654 (0.4) | 283 (0.2) | 220 (0.3) | 50 (0.2) | 11 (0.2) | ^b^ |
|  |  | 0.6 (0.5, 0.6) | 0.6 (0.5, 0.7) | 0.4 (0.3, 0.6) | 0.5 (0.3, 0.9) | ^c^ |
| Other group | 3,151 (0.1) | 78 (0.1) | 49 (0.1) | 19 (0.1) | ^b^ | ^b^ |
|  |  | 1.1 (0.9, 1.4) | 1.0 (0.8, 1.3) | 1.2 (0.7, 1.8) | 2.4 (1.1, 5.0) | ^c^ |
| Multiracial | 114,631 (2.0) | 4,806 (3.8) | 3,039 (3.4) | 1,295 (4.3) | 355 (6.6) | 117 (5.9) |
|  |  | 1.9 (1.8, 1.9) | 1.7 (1.6, 1.8) | 2.2 (2.1, 2.3) | 3.5 (3.1, 3.8) | 3.0 (2.5, 3.7) |
| White | 1,461,149 (25.6) | 56,644 (44.8) | 39,132 (43.8) | 14,051 (47.1) | 2,614 (48.8) | 847 (42.3) |
|  |  | 2.3 (2.3, 2.3) | 2.2 (2.2, 2.3) | 2.6 (2.5, 2.6) | 2.8 (2.6, 2.9) | 2.1 (2.0, 2.3) |
| **Education (years)** |  |  |  |  |  |  |
| <12 | 1,096,484 (19.2) | 12,019 (9.5) | 8,345 (9.4) | 2,366 (7.9) | 923 (17.2) | 385 (19.2) |
|  |  | 0.5 (0.5, 0.5) | 0.5 (0.5, 0.5) | 0.5 (0.4, 0.5) | 0.7 (0.6, 0.8) | 0.8 (0.7, 0.9) |
| 12 | 1,418,250 (24.8) | 30,108 (23.8) | 21,003 (23.5) | 6,787 (22.7) | 1,719 (32.1) | 599 (29.9) |
|  | Reference |  |  |  |  |  |
| >12 | 2,955,133 (51.8) | 77,667 (61.4) | 55,398 (62.0) | 18,898 (63.6) | 2,421 (45.2) | 864 (43.2) |
|  |  | 1.2 (1.2, 1.2) | 1.3 (1.2, 1.3) | 1.3 (1.3, 1.4) | 0.7 (0.6, 0.7) | 0.7 (0.6, 0.8) |
| **Payer for delivery** |  |  |  |  |  |  |
| Private | 2,703,474 (47.4) | 77,064 (60.9) | 55,478 (62.1) | 19,036 (63.8) | 1,939 (36.2) | 611 (30.5) |
|  | Reference |  |  |  |  |  |
| Public | 2,651,818 (46.4) | 44,514 (35.2) | 30,416 (34.1) | 9,687 (32.5) | 3,133 (58.5) | 1,278 (63.9) |
|  |  | 0.6 (0.6, 0.6) | 0.6 (0.6, 0.6) | 0.5 (0.5, 0.5) | 1.6 (1.6, 1.7) | 2.1 (1.9, 2.3) |
| TRICARE (Active Duty Military) | 24,625 (0.4) | 848 (0.7) | 553 (0.6) | 256 (0.9) | 30 (0.6) | ^b^ |
|  |  | 1.2 (1.1, 1.3) | 1.1 (1.0, 1.2)^a^ | 1.5 (1.3, 1.7) | 1.7 (1.2, 2.4) | 1.6 (0.8, 3.1) |
| Other | 330,129 (5.8) | 4,069 (3.2) | 2,850 (3.2) | 861 (2.9) | 255 (4.8) | 103 (5.2) |
|  |  | 0.4 (0.4, 0.5) | 0.4 (0.4, 0.4) | 0.4 (0.3, 0.4) | 1.1 (0.9, 1.2) | 1.4 (1.1, 1.7) |
| **WIC participation** |  |  |  |  |  |  |
| No | 2,801,485 (49.1) | 75,312 (59.5) | 54,091 (60.6) | 18,294 (61.3) | 2,202 (41.1) | 725 (36.2) |
|  | Reference |  |  |  |  |  |
| Yes | 2,853,058 (50.0) | 50,117 (39.6) | 34,479 (38.6) | 11,313 (37.9) | 3,090 (57.7) | 1,235 (61.7) |
|  |  | 0.7 (0.7, 0.7) | 0.6 (0.6, 0.6) | 0.6 (0.6, 0.6) | 1.4 (1.3, 1.5) | 1.7 (1.5, 1.8) |
| **Adequacy of prenatal care**^d^ |  |  |  |  |  |  |
| Adequate plus/adequate | 4,104,014 (71.9) | 92,159 (72.9) | 65,659 (73.5) | 21,915 (73.4) | 3,394 (63.4) | 1,191 (59.5) |
|  | Reference |  |  |  |  |  |
| Intermediate | 791,174 (13.9) | 20,033 (15.8) | 14,139 (15.8) | 4,811 (16.1) | 797 (14.9) | 286 (14.3) |
|  |  | 1.1 (1.1, 1.1) | 1.1 (1.1, 1.1) | 1.1 (1.1, 1.2) | 1.2 (1.1, 1.3) | 1.2 (1.1, 1.4) |
| Inadequate | 592,782 (10.4) | 11,042 (8.7) | 7,150 (8.0) | 2,512 (8.4) | 957 (17.9) | 423 (21.1) |
|  |  | 0.8 (0.8, 0.8) | 0.8 (0.7, 0.8) | 0.8 (0.8, 0.8) | 2.0 (1.8, 2.1) | 2.5 (2.2, 2.7) |
| **Other birthing person factors** |  |  |  |  |  |  |
| **Age at delivery (years)** |  |  |  |  |  |  |
| < 18 | 116,028 (2.0) | 1,143 (0.9) | 780 (0.9) | 269 (0.9) | 58 (1.1) | 36 (1.8) |
|  |  | 0.5 (0.4, 0.5) | 0.5 (0.4, 0.5) | 0.5 (0.4, 0.5) | 0.5 (0.4, 0.7) | 0.8 (0.6, 1.2) |
| 18-34 | 4,459,752 (78.1) | 94,843 (75.0) | 67,101 (75.1) | 21,746 (72.9) | 4,355 (81.3) | 1,641 (82.0) |
|  | Reference |  |  |  |  |  |
| >34 | 1,134,088 (19.9) | 30,507 (24.1) | 21,414 (24.0) | 7,825 (26.2) | 944 (17.6) | 324 (16.2) |
|  |  | 1.3 (1.2, 1.3) | 1.3 (1.2, 1.3) | 1.4 (1.4, 1.5) | 0.9 (0.8, 0.9) | 0.8 (0.7, 0.9) |
| **Parity** |  |  |  |  |  |  |
| Nulliparous | 2,217,424 (38.8) | 53,387 (42.2) | 38,155 (42.7) | 12,331 (41.3) | 2,135 (39.9) | 766 (38.3) |
|  |  | 1.1 (1.1, 1.2) | 1.2 (1.2, 1.2) | 1.1 (1.1, 1.1) | 1.0 (1.0, 1.1) | 1.0 (0.9, 1.1) |
| Multiparous | 3,487,990 (61.1) | 72,969 (57.7) | 51,050 (57.2) | 17,484 (58.6) | 3,208 (59.9) | 1,227 (61.3) |
|  | Reference |  |  |  |  |  |
| **Health conditions/exposures** |  |  |  |  |  |  |
| **Diabetes** |  |  |  |  |  |  |
| None | 5,128,592 (89.8) | 109,063 (86.2) | 77,491 (86.8) | 25,347 (84.9) | 4,589 (85.7) | 1,636 (81.8) |
|  | Reference |  |  |  |  |  |
| Gestational diabetes | 524,359 (9.2) | 14,368 (11.4) | 9,922 (11.1) | 3,613 (12.1) | 583 (10.9) | 250 (12.5) |
|  |  | 1.3 (1.3, 1.3) | 1.2 (1.2, 1.3) | 1.4 (1.3, 1.4) | 1.2 (1.1, 1.4) | 1.5 (1.3, 1.7) |
| Pre-existing diabetes | 57,095 (1.0) | 3,064 (2.4) | 1,884 (2.1) | 880 (3.0) | 185 (3.5) | 115 (5.8) |
|  |  | 2.4 (2.4, 2.5) | 2.1 (2.1, 2.2) | 3.1 (2.9, 3.3) | 3.6 (3.1, 4.2) | 6.3 (5.2, 7.6) |
| **Hypertension** |  |  |  |  |  |  |
| None | 5,235,581 (91.7) | 102,617 (81.1) | 73,070 (81.8) | 23,826 (79.9) | 4,221 (78.8) | 1,500 (75.0) |
|  | Reference |  |  |  |  |  |
| Gestational hypertension | 163,761 (2.9) | 7,105 (5.6) | 4,956 (5.6) | 1,736 (5.8) | 309 (5.8) | 104 (5.2) |
|  |  | 2.2 (2.1, 2.2) | 2.1 (2.1, 2.2) | 2.3 (2.2, 2.4) | 2.3 (2.1, 2.6) | 2.2 (1.8, 2.7) |
| Pre-existing hypertension | 70,196 (1.2) | 4,804 (3.8) | 3,207 (3.6) | 1,190 (4.0) | 265 (5.0) | 142 (7.1) |
|  |  | 3.3 (3.2, 3.4) | 3.2 (3.1, 3.3) | 3.7 (3.5, 3.9) | 4.7 (4.1, 5.3) | 7.0 (5.9, 8.4) |
| Preeclampsia | 210,871 (3.7) | 10,347 (8.2) | 6,984 (7.8) | 2,703 (9.1) | 456 (8.5) | 204 (10.2) |
|  |  | 2.3 (2.3, 2.4) | 2.2 (2.2, 2.3) | 2.6 (2.5, 2.7) | 2.5 (2.3, 2.8) | 3.1 (2.7, 3.6) |
| **Infection**^e^ | 498,859 (8.7) | 26,125 (20.7) | 16,766 (18.8) | 6,715 (22.5) | 1,724 (32.2) | 920 (46.0) |
|  |  | 2.6 (2.6, 2.7) | 2.4 (2.3, 2.4) | 3.0 (2.9, 3.1) | 4.9 (4.7, 5.2) | 8.9 (8.1, 9.7) |
| **Smoked during pregnancy**^e^ | 120,063 (2.1) | 11,776 (9.3) | 6,148 (6.9) | 3,232 (10.8) | 1,627 (30.3) | 769 (38.4) |
|  |  | 4.4 (4.4, 4.5) | 3.3 (3.2, 3.4) | 5.5 (5.3, 5.7) | 20.1 (18.9, 21.3) | 28.9 (26.4, 31.6) |
| **Drug/alcohol use during pregnancy**^e^ | 88,075 (1.5) | 12,475 (9.9) | 6,140 (6.9) | 3,619 (12.1) | 1,748 (32.6) | 968 (48.4) |
|  |  | 6.2 (6.1, 6.4) | 4.5 (4.4, 4.6) | 8.5 (8.2, 8.8) | 30.3 (28.7, 32.1) | 59.2 (54.2, 64.6) |

WIC: Special Supplemental Nutrition Program for Women, Infants, and Children

cRR: unadjusted relative risk

^a^p < 0.05

^b^Not displayed when n < 11

^c^Not calculated when n < 5

^d^Based on Kotelcheck and colleagues(29)

^e^versus no
