# Supplementary material for: Infant emergency department visits, readmission, and mortality by maternal anxiety disorder during pregnancy occurring with and without other mental health conditions: a retrospective cohort study
Source: BMC Pregnancy Childbirth. 2025 Dec 23;26:83. doi: 10.1186/s12884-025-08603-y (PMC12837128; doi:10.1186/s12884-025-08603-y)
Supplement: Supplementary file 3 — Supplementary Material 3. [file 12884_2025_8603_MOESM3_ESM.docx]

Supplemental table 2. Occurrence and risk of emergency department (ED) visit, readmission, or death within the first year of life among infants born to people with anxiety compared to without an anxiety disorder by comorbid mental health conditions: overall and by payer.

|  | **Without mental health diagnosis** | **Anxiety only** | **Anxiety and depression only** | **Anxiety and non-depression mental health disorder only** | **Anxiety, depression, and other diagnosis** |
| --- | --- | --- | --- | --- | --- |
|  | n (%) | n (%) | n (%) | n (%) | n (%) |
|  |  | cRR (95% CI) | cRR (95% CI) | cRR (95% CI) | cRR (95% CI) |
|  |  | aRR1 (95% CI) | aRR1 (95% CI) | aRR1 (95% CI) | aRR1 (95% CI) |
|  |  | aRR2 (95% CI) | aRR2 (95% CI) | aRR2 (95% CI) | aRR2 (95% CI) |
|  |  | aRR3 (95% CI) | aRR3 (95% CI) | aRR3 (95% CI) | aRR3 (95% CI) |
| **Sample - all** | 5,710,046 | 89,297 | 29,840 | 5,357 | 2,001 |
| **First Year Infant Outcomes^a^** |  |  |  |  |  |
| ED visit | 1,688,197 (29.6) | 24,491 (27.4) | 7,517 (25.2) | 1,836 (34.3) | 673 (33.6) |
|  |  | **0.93 (0.92, 0.94)** | **0.85 (0.83, 0.87)** | **1.16 (1.11, 1.21)** | **1.14 (1.05, 1.23)** |
|  |  | **0.93 (0.91, 0.94)** | **0.84 (0.83, 0.87)** | **1.15 (1.10, 1.21)** | **1.13 (1.05, 1.22)** |
|  |  | 1.00 (0.99, 1.02) | **0.94 (0.92, 0.96)** | **1.09 (1.04, 1.14)** | 1.02 (0.95, 1.10) |
|  |  | 0.99 (0.98, 1.01) | **0.92 (0.90, 0.94)** | 1.04 (0.99, 1.09) | 0.97 (0.90, 1.05) |
| Readmission | 528,659 (9.3) | 6,991 (7.8) | 2,231 (7.5) | 532 (9.9) | 223 (11.1) |
|  |  | **0.85 (0.83, 0.87)** | **0.83 (0.80, 0.86)** | 1.07 (0.98, 1.17) | **1.20 (1.06, 1.37)** |
|  |  | **0.83 (0.81, 0.85)** | **0.78 (0.74, 0.82)** | 1.03 (0.95, 1.12) | 1.11 (1.00, 1.29) |
|  |  | **0.87 (0.85, 0.89)** | **0.83 (0.80, 0.87)** | 1.01 (0.92, 1.10) | 1.09 (0.95, 1.24) |
|  |  | **0.86 (0.84, 0.88)** | **0.81 (0.78, 0.85)** | 0.96 (0.88, 1.04) | 1.00 (0.88, 1.15) |
| Death | 16,484 (0.3) | 387 (0.4) | 151 (0.5) | 32 (0.6) | 22 (1.1) |
|  |  | **1.50 (1.36, 1.66)** | **1.75 (1.49, 2.06)** | **2.07 (1.46, 2.93)** | **3.81 (2.51, 5.79)** |
|  |  | **1.25 (1.13, 1.38)** | **1.33 (1.13, 1.56)** | **1.47 (1.04, 2.08)** | **2.32 (1.53, 3.53)** |
|  |  | **1.29 (1.16, 1.42)** | **1.37 (1.17, 1.61)** | 1.25 (0.89, 1.78) | **1.89 (1.25, 2.88)** |
|  |  | **1.18 (1.07, 1.31)** | **1.19 (1.01, 1.39)** | 0.92 (0.65, 1.31) | 1.23 (0.81, 1.87) |
| **Sample – Public payer** | 2,696,332 | 30,416 | 9,687 | 3,133 | 1,278 |
| **First Year Infant Outcomes^a^** |  |  |  |  |  |
| ED visit | 1,036,390 (39.1) | 12,247 (40.3) | 3,550 (36.7) | 1,223 (39.0) | 459 (35.9) |
|  |  | **1.03 (1.01, 1.05)** | **0.94 (0.91, 0.97)** | 1.00 (0.94, 1.06) | 0.92 (0.84, 1.01) |
|  |  | **1.03 (1.01, 1.05)** | **0.94 (0.91, 0.97)** | 1.00 (0.94, 1.05) | 0.92 (0.84, 1.00) |
|  |  | **1.04 (1.02, 1.06)** | **0.96 (0.93, 0.99)** | 1.01 (0.96, 1.07) | 0.92 (0.84, 1.01) |
|  |  | **1.03 (1.01, 1.05)** | **0.94 (0.91, 0.98)** | 0.99 (0.93, 1.05) | **0.91 (0.83, 0.99)** |
| Readmission | 289,731 (10.9) | 3,178 (10.5) | 990 (10.2) | 343 (11.0) | 152 (11.9) |
|  |  | **0.96 (0.92, 0.99)** | **0.94 (0.88, 1.00)** | 1.00 (0.90, 1.11) | 1.09 (0.93, 1.28) |
|  |  | **0.94 (0.91, 0.97)** | **0.91 (0.85, 0.96)** | 1.00 (0.87, 1.07) | 1.03 (0.88, 1.21) |
|  |  | **0.96 (0.92, 0.99)** | **0.93 (0.87, 0.99)** | 1.00 (0.90, 1.11) | 1.06 (0.91, 1.25) |
|  |  | **0.94 (0.90, 0.97)** | **0.90 (0.85, 0.96)** | 0.94 (0.85, 1.05) | 0.99 (0.84, 1.16) |
| Death | 9,221 (0.4) | 165 (0.5) | 74 (0.8) | 21 (0.7) | 16 (1.3) |
|  |  | **1.56 (1.34, 1.82)** | **2.20 (1.75, 2.76)** | **1.93 (1.26, 2.96)** | **2.60 (2.20, 5.88)** |
|  |  | **1.28 (1.09, 1.49)** | **1.59 (1.26, 1.99)** | 1.35 (0.88, 2.07) | **2.24 (1.37, 3.65)** |
|  |  | **1.17 (1.00, 1.36)** | **1.42 (1.13, 1.78)** | 1.09 (0.71, 1.68) | **1.79 (1.10, 2.93)** |
|  |  | 1.04 (0.89, 1.21) | 1.17 (0.93, 1.47) | 0.80 (0.52, 1.23) | 1.18 (0.72, 1.94) |
| **Sample – not public payer** | 3,058,228 | 58,881 | 20,153 | 2,224 | 723 |
| **First Year Infant Outcomes^a^** |  |  |  |  |  |
| ED visit | 651,807 (21.3) | 12,244 (20.8) | 3,967 (19.7) | 613 (27.6) | 214 (29.6) |
|  |  | **0.98 (0.96, 0.99)** | **0.92 (0.90, 0.95)** | **1.29 (1.19, 1.40)** | **1.39 (1.21, 1.59)** |
|  |  | **0.97 (0.95, 0.99)** | **0.92 (0.89, 0.95)** | **1.29 (1.19, 1.39)** | **1.37 (1.20, 1.57)** |
|  |  | **0.97 (0.95, 0.98)** | **0.91 (0.88, 0.93)** | **1.16 (1.07, 1.26)** | **1.19 (1.04, 1.36)** |
|  |  | **0.96 (0.94, 0.98)** | **0.89 (0.86, 0.92)** | **1.12 (1.03, 1.21)** | 1.11 (0.97, 1.27) |
| Readmission | 238,928 (7.8) | 3,813 (6.5) | 1,241 (6.2) | 189 (8.5) | 71 (9.8) |
|  |  | **0.83 (0.80, 0.86)** | **0.79 (0.75, 0.83)** | 1.09 (0.94, 1.25) | 1.26 (1.00, 1.59) |
|  |  | **0.81 (0.79, 0.84)** | **0.76 (0.72, 0.81)** | 1.05 (0.91, 1.21) | 1.18 (0.94, 1.49) |
|  |  | **0.81 (0.79, 0.84)** | **0.76 (0.72, 0.81)** | 1.02 (0.89, 1.18) | 1.13 (0.90, 1.43) |
|  |  | **0.81 (0.78, 0.84)** | **0.75 (0.71, 0.80)** | 0.98 (0.85, 1.13) | 1.04 (0.82, 1.31) |
| Death | 7,263 (0.2) | 222 (0.4) | 77 (0.4) | 11 (0.5) | ^b^ |
|  |  | **1.59 (1.39, 1.81)** | **1.61 (1.29, 2.01)** | **2.08 (1.15, 3.76)** | **3.49 (1.57, 7.78)** |
|  |  | **1.32 (1.15, 1.50)** | 1.25 (1.00, 1.57) | 1.57 (0.87, 2.83) | 2.17 (0.97, 4.83) |
|  |  | **1.34 (1.17, 1.53)** | 1.16 (0.97, 1.41) | 1.44 (0.80, 2.60) | 1.85 (0.83, 4.13) |
|  |  | **1.28 (1.12, 1.47)** | 1.15 (0.92, 1.45) | 1.10 (0.65, 2.16) | 1.28 (0.57, 2.88) |

**Bold when p < 0.05**

cRR: unadjusted relative risk

aRR1: adjusted for preterm birth

aRR2: adjusted for preterm birth, racial/ethnicity group, education, payer (when applicable), WIC participation, adequacy of prenatal care, birthing person age, and parity

aRR3: adjusted for preterm birth, racial/ethnicity group, education, payer (when applicable), WIC participation, adequacy of prenatal care, birthing person age, parity, gestational diabetes, gestational diabetes, preexisting HTN, gestational HTN, preeclampsia, infection, smoking, drug/alcohol use diagnosis

^a^ All yes vs no

^b^not presented when n < 11
